# Promising Glaucoma Medication: A Comprehensive Translational Evaluation

**DOI:** 10.3390/pharmaceutics18070822

**Published:** 2026-07-02

**Authors:** Doaa Nabih Maria, Mohamed Moustafa Ibrahim, Sara N. Maria, Monica M. Jablonski

**Affiliations:** 1 Department of Ophthalmology, Hamilton Eye Institute, University of Tennessee Health Science Center, Memphis, TN 38163, USA; dmaria@uthsc.edu (D.N.M.); mibrahi2@uthsc.edu (M.M.I.); smaria1@uthsc.edu (S.N.M.); 2 Department of Pharmaceutics, Faculty of Pharmacy, Mansoura University, Mansoura 35516, Egypt; 3 Department of Pharmaceutical Sciences, University of Tennessee Health Science Center, Memphis, TN 38163, USA

**Keywords:** enhanced delivery formulation, glaucoma, ex vivo transcorneal permeability, in vivo bioadhesion, non-human primates

## Abstract

**Background/Objectives:** Despite available treatment options, glaucoma continues to be a leading cause of irreversible blindness. Current medications have multiple limitations, including rapid drainage, ocular irritation, requirement for multiple daily dosings, and systemic side effects. The current study was designed to engineer and characterize a pregabalin-containing enhanced delivery formulation (PRG-EDF) to directly address these inadequacies. **Methods:** PRG-EDF eye drops were prepared using ingredients that are either U.S. Food and Drug Administration (FDA)-approved for ophthalmic use or have established safety profiles. The formulation was characterized using multiple evaluations, including pH, zetasizer analyses, viscosity, in vitro drug release, transcorneal permeability, determination of dose concentration and volume, systemic exposure, and potential for tachyphylaxis. Efficacy was evaluated using both Dutch belted rabbits and baboons. **Results:** PRG-EDF provides extended release for up to 24 h. Ex vivo data reveal that PRG-EDF does not alter the inherent high PRG corneal permeability. An intraocular pressure (IOP) study using DB rabbits demonstrates that 40 µL of PRG-EDF, 0.6%, is the optimum dose of our formulation. Comparison of the efficacy of PRG-EDF with commercial products demonstrated its superiority in overall IOP-lowering efficacy. An extended in vivo assessment demonstrated that the potency of PRG-EDF reached maximum IOP-lowering amplitude after 4 weeks of daily dosing. Moreover, an in vivo bioadhesion assay demonstrated that EDF remained on the ocular surface for up to 24 h. Impressively, PRG-EDF is as effective in baboons as in rabbits. **Conclusions:** We have successfully engineered a highly promising once-daily glaucoma medication with superior efficacy, as illustrated by higher IOP-lowering ability and prolonged duration of action.

## 1. Introduction

Primary open-angle glaucoma (POAG) is the most common form of glaucoma and is considered to be the leading cause of irreversible blindness [1]. Topical intraocular pressure (IOP)-lowering eye drops are the standard of care for POAG; however, all current glaucoma medications share significant limitations. Despite the availability of several FDA-approved IOP-lowering eye drop choices, there are in fact a limited number of these being used clinically. This effectively narrows options when tolerance builds, and a patient is switched to a different drug class and/or another drug is added to their daily dosing regimen. Hence, additional therapies are needed due to tachyphylaxis and/or adverse events. Marketed drug classes include prostaglandin analogs (e.g., latanoprost) [2], beta-blockers (e.g., timolol) [3], alpha-2 adrenergic agonists (e.g., brimonidine) [4], and carbonic anhydrase inhibitors (e.g., dorzolamide) [5]. However, these glaucoma medications are not universally effective in decreasing IOP and have been associated with multiple side effects. During the current decade, two long-acting medications have received FDA approval for POAG treatment as once-daily eye drops. Unfortunately, both medications are associated with serious adverse events [6]. The first is latanoprostene bunod, a dual mechanism glaucoma medication—nitric oxide producer and a prostaglandin analog—that increases aqueous humor outflow [7]. This medication is associated with intolerable side effects such as local eye irritation (11.5%), growth of eyelashes (16.2%), increased iris pigmentation, and color change (9%) [8,9]. The second recently approved medication is netarsudil, a Rho kinase enzyme inhibitor, which increases the aqueous humor outflow and decreases its production [10]. This drug is also associated with severe adverse events that include conjunctival hyperemia (53%), conjunctival hemorrhage (20%), eye pain upon instillation (20%), and cornea verticillata (20%) [8]. Accordingly, all current glaucoma medications contribute to poor patient compliance, estimated to be as low as 40%. Therefore, visual deterioration due to glaucoma is frequent, resulting in visual field loss in millions of people around the world [11,12].

To directly address all the above-listed pain points and unmet needs, we previously identified a new druggable target for the treatment of glaucoma, as well as a highly promising drug and formulation. Several studies recently published from our lab demonstrated that pregabalin (PRG) can target CACNA2D1 (α2δ1) with high selectivity and high affinity to lower the IOP in various preclinical models, including several mouse strains and Dutch belted rabbits [13,14,15]. PRG represents a first-in-class glaucoma medication that acts through a novel, previously unknown target to lower IOP, thus providing a promising and much-needed option for many patients. Because α2δ1 is localized to both aqueous humor (AH) production and AH conventional drainage structures of the eye [13,14], it is possible that PRG lowers IOP through reducing AH production or increasing AH drainage, or both, although the precise mechanism of action has not been delineated.

While our previously developed sustained-release, bioadhesive, once-daily PRG-loaded microemulsion eye drops were a promising treatment for POAG, the inactive ingredients used for its production were of research chemical grade [14,15]. Hence, these previously developed PRG microemulsion eye drops are not suitable to be used in clinical trials during a pathway toward FDA approval as once-daily glaucoma eye drops.

The purpose of the current studies was to engineer a revised version of long-acting PRG eye drops with extended drug release and prolonged corneal contact time using safe ingredients that are listed in the FDA-approved inactive ingredient list for ophthalmic use and/or excipients that are reported as safe. Furthermore, we sought to test this current version formulation to determine if it is equally as effective as our published microemulsion. Our PRG-EDF eye drops were characterized using numerous in vitro, ex vivo, and in vivo evaluations. By carefully selecting excipients, including a bioadhesive polymer, and optimizing the applied ocular dose in preclinical studies, we demonstrate the superiority of our bioadhesive PRG-EDF, 0.6%, in comparison to several commercially available glaucoma medications. Further, PRG-EDF, 0.6%, demonstrated excellent efficacy with a ~27% reduction in IOP after a single dose in two animal models, DB rabbits and non-human primates, thereby providing a positive bridging study for future clinical trials in humans.

## 2. Materials and Methods

### 2.1. Materials

Pregabalin (PRG) was purchased from Nantong Chanyoo Pharmatech Co., Ltd. (Nantong, China). Magnesium chloride hexahydrate, dextrose, sodium chloride, phosphoric acid, dimethyl sulfoxide, formic Acid (99+%, Optima™ LC/MS Grade), methanol (HPLC grade), acetonitrile (HPLC grade), and methaqualone solution (1.0 mg/mL in methanol) were purchased from Fisher Scientific (Fair Lawn, NJ, USA). Soybean L-α-Lecithin was obtained from Calbiochem (Billerica, MA, USA). Glycol chitosan (GCS, ≥60% Purity) was procured from Santa Cruz Biotechnology, Inc., Dallas, TX, USA. Ethyl alcohol was obtained from Decon Labs, Inc. (King of Prussia, PA, USA). Potassium chloride, dipotassium hydrogen orthophosphate, acridine orange hydrochloride, calcium chloride dihydrate, glutathione disulfide, sodium phosphate dibasic, sodium bicarbonate, and potassium dihydrogen phosphate were purchased from Sigma-Aldrich (St. Louis, MO, USA). Fresh eyes of New Zealand white rabbits were purchased from Pel-Freez Biologicals (Rogers, AR, USA). Crodamol GTCC, sorbitan sesquioleate (Span 83), super refined (SR) Brij O2, SR polysorbate 20, SR P35 Castor oil, and SR polyethylene glycol (PEG400) were purchased from Croda Inc. (Plainsboro, NJ, USA). Water for injection (WFI) was purchased from Corning (Manassas, VA, USA).

### 2.2. Animals

Dutch belted rabbits (DB rabbits; equal numbers of males and females) were purchased from Covance Inc. (Princeton, NJ, USA), weighed ~1.6–2.4 kg, and were aged 12–18 months. Non-human primate (NHP) species, Papio Anubis (olive baboon), or mixed Papio anubis and Papio cynocephalus (yellow baboon) were procured from the Texas Biomedical Research Institute (San Antonio, TX, USA). Seven female baboons were used, aged 9–13 years and weighing 13–22 kg. All procedures including DB rabbits and baboons were approved by the Use Review Board and the Animal Care of the University of Tennessee Health Science Center (UTHSC) and complied with the Association of Research in Vision and Ophthalmology (ARVO) Statement for the Use of Animals in Ophthalmic and Vision Research, in addition to the guidelines for laboratory animal experiments (Institute of Laboratory Animal Resources, Public Health Service Policy on Humane Care and Use of Laboratory Animals).

### 2.3. Pregabalin Assay

To quantify PRG within in vitro and ex vivo evaluations, a previously published reversed-phase HPLC-UV method was used [16]. A Supelco Kromasil C18 column (5 µm, 100 °A, 4.0 mm × 300 mm) was used with an Agilent 1100 series HPLC system (Waldbronn, Germany). A mixture of methanol:acetonitrile: 0.02 M dipotassium hydrogen orthophosphate (3:1:16, *v*/*v*/*v*) was used as the mobile phase at a flow rate of 1 mL/min. A photodiode array detector monitored the effluent, and PRG was detected at 210 nm after 5.4 min.

Quantification of PRG in biological samples used the ultra-high-performance liquid chromatography-tandem mass spectrometry (UPLC–MS/MS) method that was developed and validated by Pauly et al. (2013) [17] and verified in our laboratory. The limit of quantification (LOQ) was 2.5 pg/mg, and the limit of detection (LOD) was 0.76 pg/mg.

### 2.4. Preparation of PRG-Enhanced Delivery Formulation (EDF)

To produce a safe and well-tolerated formulation, PRG-EDF, a multiple microemulsion, was engineered by replacing several ingredients of our previously developed PRG microemulsion, PRG-ME [14,15] with other ingredients that are certified safe for ophthalmic use. The PRG-EDF formulation components and their ratios were carefully selected using our previously published formulation [14,15] as a baseline. An equivalent FDA-approved surfactant with a similar hydrophilic–lipophilic balance (HLB) value replaced each surfactant from the original PRG-ME formulation (Table 1) [14,15]. Furthermore, glycol chitosan was selected to be used as a bioadhesive polymer in our PRG-EDF formulation to support its once-daily application.

**Figure 1:**
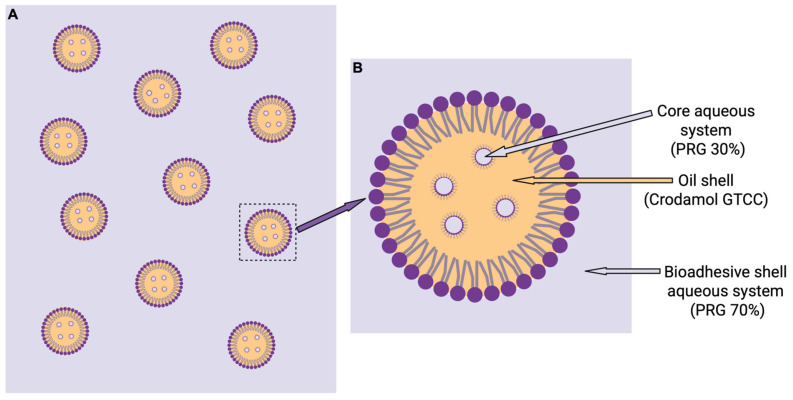
(**A**) Cartoon of PRG-EDF, 0.6%, demonstrating several droplets of w/o emulsion scattered within the bioadhesive shell aqueous system. (**B**) A magnified droplet of the PRG-EDF, 0.6%, consists of several nuclei of the core aqueous system entrapped in the oil phase, which is surrounded by the bioadhesive shell aqueous system. Created in BioRender. Maria, D. (2026) https://BioRender.com/7o4nnqv.

In our previously published PRG-ME eye drops, 0.6% PRG was the minimum concentration required to achieve the maximum reduction in IOP [15]. While we expect that our newly engineered PRG-EDF will demonstrate a similar efficacy profile, this must be empirically evaluated. Hence, we prepared and evaluated PRG-EDF eye drops using different concentrations of PRG for further evaluation of their in vivo efficacy. PRG was incorporated into the EDF at four concentrations (*w*/*w*): 0.4; 0.5; 0.6; and 0.7%. The EDF was prepared in several steps. Briefly, Crodamol GTCC (oil phase) was mixed with a hydrophobic surfactant mixture, Span 83:SR Brij O2:Lecithin (3:3:4). WFI containing 30% of PRG was added to the previously prepared oil/surfactant mixture to compose the core aqueous system inside an oil shell (w/o emulsion). The second step included the bioadhesive shell aqueous system preparation by incorporating the bioadhesive polymer, glycol chitosan (GCS), in a mixture of WFI containing 70% of PRG and hydrophilic surfactants and cosurfactants, SR polysorbate 20:SR P35 Castor oil:SR PEG400 (2:2:6). The last step entailed the simple mixing of the prepared w/o emulsion with the polymeric bioadhesive shell aqueous system to produce the final EDF microemulsion eye drops (Figure 1). Blank EDF eye drops were prepared using the same method without the addition of PRG.

### 2.5. Exploratory In Vivo Studies

#### 2.5.1. Drop Volume Response Study

This study was performed to determine the effect of the drop volume on the IOP-lowering efficacy of PRG-EDF, 0.6% (this concentration was selected based on our previously published PRG-ME formulation) [14,15]. These data are essential to determine the most effective drop volume, taking into consideration the maximum allowable drop size that can be applied to the human eye. Three different drop volumes were evaluated: 30; 40; and 50 μL. Each dose volume was applied bilaterally into the inferior conjunctival sac of both eyes of DB rabbits (*n* = 6, 12 eyes), using our previously published protocol [13,14,15]. DB rabbits were selected to evaluate the IOP-lowering efficacy of the prepared formulations as an animal model because their IOP is spontaneously elevated post-puberty, which supports their use as a preclinical model to determine the efficacy of an IOP-lowering medication [18,19,20,21,22]. IOP was measured before treatment as a baseline, followed by predetermined time intervals after application up to 30 h post-dosing using a Tono-Pen AVIA Vet (Reichert Technologies, Depew, NY, USA). For comparison between the different drop volumes, various pharmacodynamic (PD) parameters were calculated using GraphPad Prism 10 software, including maximum reduction in IOP calculated as a percentage from the baseline (% IOP reduction), time required to reach maximum reduction in IOP (T_max_), time required for IOP to return to baseline value (i.e., end of drug effect; T_end_), and total area under the % IOP-vs-time curve (AUC). The data were statistically analyzed using one-way analysis of variance (ANOVA) followed by Tukey’s multiple comparisons test. Results were reported as mean ± SEM.

#### 2.5.2. Dose Response and EC_50_ Determination Study

PRG-EDF eye drops were prepared according to the previously mentioned method using four different PRG concentrations (0.4%, 0.5%, 0.6%, and 0.7% *w*/*w*). A single dose-response design was used to evaluate each formulation. Based on the outcomes of our drop volume response study, 40 μL of each PRG-EDF eye drops were applied into the inferior conjunctival sac of both eyes of DB rabbits (*n* = 6 rabbits, 12 eyes) using our previously published protocol [13,14,15]. IOP was measured before treatment (baseline) and at predetermined time intervals after application up to 30 h post-dosing using a Tono-Pen AVIA Vet (Reichert Technologies, Depew, NY, USA). For comparison of PRG IOP-lowering efficacy of different PRG concentrations, PD parameters were calculated, and statistically significant differences were determined as listed above. To determine the EC_50_ of PRG as an IOP-lowering drug, the AUC of the % IOP reduction versus time curve for each PRG formulation—0.4%, 0.5%, 0.6%, and 0.7% *w*/*w*—against the corresponding PRG concentration was plotted. The EC_50_ was calculated by extrapolation as the PRG concentration corresponding to 50% of the largest IOP-lowering response [15].

### 2.6. In Vitro Characterizations of PRG-EDF

#### 2.6.1. Measurement of pH

The pH values of the blank and the medicated EDF eye drops were measured using a pH meter (Corning pH meter 440; Corning Inc., Corning, NY, USA). One gram of each formulation was dispersed in 20 mL of Milli-Q water, and the pH was measured. The pH was measured in triplicate, and the results were calculated as mean ± SEM.

#### 2.6.2. Measurement of Average Droplet Size, Polydispersity Index (PDI), and Zeta Potential

After suitable dilution, the average droplet size, PDI, and zeta potential of PRG-EDF, 0.6% eye drops were determined using Zetasizer (Nanoseries, nano-ZS, Malvern Instruments Limited, Malvern, UK). The droplet size and PDI were measured by Dynamic Light Scattering at a backscatter angle of detection (173°) at the exact center of the cell at a count rate of 270 kcps. The zeta potential was measured using Electrophoretic Light Scattering (ELS) at a forward angle of 13° degrees at the exact center of the cell at a count rate of 300 kcps. All measurements were performed at 25 °C. Three independent test run results were calculated as mean ± SEM.

#### 2.6.3. Determination of Viscosity

The viscosity of both blank and medicated EDF was measured using a Discovery Hybrid Rheometer DHR-3 (Waters TA Instruments, New Castle, DE, USA) at 5°, 25°, and 35 °C, using the flow rheological technique according to our previously published protocol [23]. Viscosity was determined using a cone and plate attachment over a range of shear rates from 1 to 1000 s^−1^ [24]. All measurements were performed in triplicate to confirm accuracy and consistency, and the results were expressed as the mean ± SEM. Viscosity data were statistically analyzed using a one-way analysis of variance (ANOVA) test, followed by Tukey’s multiple comparisons test.

#### 2.6.4. In Vitro Drug Release

The in vitro PRG release behavior from the prepared PRG-EDF, 0.6%, was performed according to our previously published protocol [13,14]. Fast microequilibrium dialyzers (1500 μL) to which a semipermeable regenerated cellulose membrane was attached were used (molecular weight cutoff of 5000 Da, Harvard Apparatus Co., Holliston, MA, USA). Forty microliters (this dose volume was selected based on the results of the exploratory dose volume study) of PRG-EDF, 0.6%, or controls—PRG aqueous solution, 0.6%, and PRG in GCS polymeric solution, 0.6%—was placed in the donor chamber. In the receptor chamber, warmed PBS pH 7.4 (1.5 mL at 35 ± 0.5 °C) was placed. The dialyzer was kept in a thermostatically controlled shaker (35 ± 0.5 °C and 50 rpm). The entire medium in the receptor chamber was withdrawn at predetermined time intervals ranging from 0.25 to 24 h and replaced with 1.5 mL of fresh, warmed medium. The concentration of the released PRG was determined by the HPLC-UV assay method. The cumulative percent amount released of PRG was calculated as mean ± SEM. All experiments were performed in triplicate. To study the release kinetics of PRG from PRG-EDF, 0.6%, as well as the two controls, the release data were fitted to different release kinetics models—zero order, first order, Higuchi, and Korsmeyer–Peppas—to determine the model that best fit our release data [25,26,27]. The data were analyzed for statistical significance using one-way analysis of variance (ANOVA) followed by Tukey’s multiple comparisons test. Kinetics and statistical analysis were determined using GraphPad Prism 10 software (GraphPad Software, Inc., San Diego, CA, USA).

### 2.7. Ex Vivo Transcorneal Permeability

To determine the transcorneal permeability of PRG from PRG-EDF, 0.6% eye drops, as well as from the control formulation, PRG aqueous solution, 0.6%, we used fresh rabbit corneas excised from the whole eyes of New Zealand white rabbits that were shipped overnight from Pel-Freez Biologicals LLC (Rogers, AR, USA) in Hanks balanced salt solution over wet ice. Modified rounded junction Franz diffusion cells (PermeGear Inc., Allentown, PA, USA) were used, wherein corneas were mounted to the cells with the epithelial side facing up toward the donor chamber containing the formulations and the endothelial side facing down toward the receptor chamber containing balanced salt solution (BSS-Plus) [28]. The temperature of the cells was maintained at 35 ± 0.5 °C with the aid of a circulating water bath. Forty microliters of PRG-EDF, 0.6% eye drops, or PRG aqueous solution, 0.6%, were placed in the donor chambers. The receptor chambers were continuously stirred and filled with 5 mL of BSS-Plus (Alcon Laboratories Inc., Fort Worth, TX, USA), which has a similar composition to aqueous humor and is commonly used as a sterile intraocular irrigating solution due to its ability to maintain the anatomic and physiologic integrity of intraocular tissues [29,30]. An amount of 500 μL was withdrawn from the receptor chamber at predetermined time intervals (1, 2, 3, 4, 5, 6, 7, and 8 h) and replaced with an equal volume of prewarmed BSS-Plus. Using the HPLC-UV assay method, the drug concentration in the withdrawn samples was immediately determined. The results were plotted as cumulative amount permeated (μg) versus time. The steady-state flux (*J*) was calculated by dividing the rate of permeation by the surface area of the cornea through which permeation occurred, while the permeability coefficient (*P*) was calculated by dividing the flux by the initial drug concentration in the donor chamber according to the following equations [28]:

(1) Flux (J)=(dM÷dt)÷A

(2) $$Permeability\;(P)=Flux÷C_{d}$$

where *dM*/*dt* (the rate of permeation) is the slope of the straight line obtained after plotting the cumulative amount permeated versus time, (*A*) is the surface area of the corneal membrane through which the drug is permeated, which is equal to 0.636 cm^2^, and (*C_d_*) is the initial drug concentration in the donor chamber. Four replicates were conducted for each formulation, the results were calculated as mean ± SEM, and the data were statistically analyzed by unpaired *t*-test using GraphPad Prism 10 software.

### 2.8. Pregabalin-EDF In Vivo Evaluations

#### 2.8.1. Comparison of the IOP-Lowering Efficacy of PRG-EDF, 0.6%, with Marketed Glaucoma Medications

The IOP-lowering effect of PRG-EDF, 0.6%, was compared to three commercially available IOP-lowering medications: timolol maleate, 0.5% (Alcon Laboratories, Inc., Fort Worth, TX, USA); latanoprost, 0.005% (Bausch & Lomb, Tampa, FL, USA); and latanoprostene bunod, 0.024% (Bausch & Lomb, Bridgewater, NJ, USA). A single dose of 40 µL of each IOP-lowering medication, as well as PRG-EDF, 0.6%, was applied into the inferior conjunctival sac of both eyes of DB rabbits (*n* = 6 rabbits, 12 eyes). IOP was measured using a Tono-Pen AVIA Vet according to our previously published protocol [13,14,15] before treatment (baseline) and at predetermined time intervals after application up to 30 h post-dosing. Pharmacodynamic parameters were calculated and statistically analyzed by one-way analysis of variance (ANOVA) test followed by Tukey’s multiple comparisons test using GraphPad Prism 10 software. Results were reported as mean ± SEM.

#### 2.8.2. Exploratory Plasma Pharmacokinetic (PK) Study After Topical Ocular Administration of a Single Dose of PRG-EDF, 0.6%

To evaluate PRG PK in plasma after a single topical application of PRG-EDF, 0.6%, six DB rabbits (3 males and 3 females) were used. A single drop of 40 μL PRG-EDF, 0.6%, was bilaterally applied into the inferior conjunctival sac of DB rabbit eyes. A total of 0.3 mL of blood was collected from each rabbit through the marginal ear vein at predetermined time intervals of 0, 1, 2, 3, 4, 5, 6, 8, 24, and 48 h. Blood was immediately separated into plasma and stored at −80 °C until the time of processing. All plasma samples were assayed using the LC-MS/MS method [17]. Standard curves were constructed using the same procedures on naïve DB rabbit plasma spiked with known concentrations of PRG that ranged from 1 to 1000 ng/mL.

#### 2.8.3. Long-Term Efficacy Study: 30 Days of a Single Daily Application of PRG-EDF, 0.6% in DB Rabbits

To directly address potential tachyphylaxis, which is frequently observed with glaucoma eye drops, the efficacy of the PRG-EDF, 0.6% eye drops, upon prolonged use was determined. IOP was measured in DB rabbits (*n* = 6 rabbits, 12 eyes) dosed once daily for 30 consecutive days bilaterally with 40 μL of PRG-EDF, 0.6%. The IOP of both eyes was measured twice daily, immediately before the formulation application and at the time of maximum IOP reduction (T_max_). Using GraphPad Prism 10 software, the % IOP reduction was calculated and plotted against the corresponding time over the 30-day study. Results were reported as mean ± SEM.

#### 2.8.4. IOP-Lowering Efficacy of PRG-EDF, 0.6% in Non-Human Primates After a Single Topical Application

The IOP-lowering effect of the PRG-EDF, 0.6%, was determined using baboons (non-human primates; NHPs) (*n* = 7 female baboons, 14 eyes). Ketamine, 10 mg/kg, was used to sedate the animals during dosing and IOP measurements. 40 μL of the PRG-EDF, 0.6% eye drops, was bilaterally applied into the inferior conjunctival sac of all baboon eyes. The IOP was measured using a Tono-pen AVIA (Reichert Technologies, Depew, NY, USA) immediately before the application of the formulation as a baseline and at predetermined time intervals (4, 6, 8, and 24 h). Five consecutive IOP readings were averaged for each eye at each measurement. The PD parameters were calculated using GraphPad Prism 10 software as mentioned above. The results were presented as mean ± SEM.

#### 2.8.5. Bioadhesion Study of EDF on the Corneal Surface of DB Rabbits

To evaluate the ability of PRG-EDF to adhere to the eye surface after a single topical application, EDF was prepared using an autofluorescent cargo, allowing for easy visualization. Acridine orange (AO), a water-soluble fluorescent dye, was incorporated into the EDF at a concentration of 0.6% *w*/*w*, which is equivalent to that in PRG-EDF. The acridine orange molecule was selected because it has physicochemical properties similar to those of PRG (both are Biopharmaceutics Classification System (BCS) class I with high aqueous solubility, good permeability, and have close molecular weight values) [15]. DB rabbits (*n* = 5) received 40 μL of the fluorescent EDF in one eye while the other eye received 40 μL of aqueous solution of the fluorescent dye, 0.6% *w*/*w* (AO in PBS). Rabbit eyes were photographed using a ClearView-2 camera (Optibrand, Fort Collins, CO, USA), and tear samples were collected immediately before the application and at predetermined time points—5 min, 1, 6, 12, and 24 h—after application of the formulations. For quantification of the fluorescent intensity, rabbit eye photos were evaluated for the fluorescent intensity using ImageJ software. Tear samples were assayed spectrophotofluorometrically to quantify their AO content using a UV/fluorescence microplate reader spectrophotofluorometer (μ-Quant Bio-Tek Instruments, Inc., Winooski, VT, USA) at excitation/emission wavelengths of 490/520 nm. The AO concentrations were calculated from the standard curve. The results were presented as mean ± SEM.

## 3. Results and Discussion

### 3.1. Preparation of PRG-Enhanced Delivery Formulation (EDF)

All ratios and combinations of oil, surfactants, and WFI used to prepare the current version of PRG-EDF, 0.6% eye drops, were selected based on the results of a preliminary study (Table 1), wherein each surfactant in the previous version of PRG-ME formulation has been replaced by an equivalent surfactant (s) of similar HLB value. Because PRG is a BCS class I drug, it is highly water-soluble and exhibits rapid washout from the corneal surface when applied topically in the form of an aqueous solution, resulting in poor ocular bioavailability (<5%) [14]. Therefore, the use of a bioadhesive polymer is one of the successful approaches to achieve a longer corneal residence time of topically applied eye drops, thereby enhancing their bioavailability and reducing the administration frequency associated with better patient compliance and acceptance. We carefully selected GCS as a water-soluble derivative of chitosan because of its many advantages over the parent chitosan. Chitosan is a linear, positively charged natural polysaccharide polymer derived from chitin [31]. It has been widely used in the biomedical field due to its safety, biocompatibility, bioadhesion, and biodegradability [32]. However, the parent chitosan molecule is typically insoluble in water and soluble only at an acidic pH [32]. Fortunately, glycol chitosan (GCS) is a water-soluble chitosan derivative [33,34]. Chitosan itself is reported in the GRAS (Generally Recognized as Safe) material list. Interestingly, GCS was reported to be safer than chitosan because of its poor immunogenic effect compared to chitosan [35,36]. Moreover, GCS advantages include biocompatibility, biodegradability, bioadhesiveness, water solubility, and low cytotoxicity [34].

In addition to the selection of the bioadhesive polymer, other excipients were also selected to be either FDA-approved for ophthalmic use or reported as safe ingredients [37]. Crodamol GTCC is a biodegradable medium-chain triglyceride of caprylic (C_8_) and capric (C_10_) saturated fatty acids. This oil was selected due to the absence of unsaturated fatty acids, which allows the oil to resist rancidity and help to improve the product stability [15]. Because PRG is a BCS class I molecule, it is highly soluble in WFI to form the inner aqueous core. In contrast, PRG is completely insoluble in Crodamol GTCC. Thus, Crodamol GTCC was selected to be the oil phase of PRG-EDF, 0.6% eye drops, where it supported the required sustained drug release. A mixture of three surfactants—Span 83:SR Brij O2:Lecithin (3:3:4)—was added to the Crodamol GTCC to form the hydrophobic shell around the aqueous core in which the drug was incorporated. This hydrophobic barrier hinders drug release, which contributes to the sustained release property of the formulation for such a water-soluble drug. Lecithin was selected as a surfactant in our EDF eye drops because it has many advantages, including its biosafety, biodegradability, biocompatibility, and natural occurrence, making it a well-tolerated surfactant [38,39]. Furthermore, because of its biodegradability and its phospholipid composition that resembles the structure of the biological cell membranes, it is regarded as an ideal biological surfactant [40]. Moreover, these oily excipients tend to prevent ocular surface dryness by helping to restore the lipid layer of the tear film [41].

The outer aqueous shell of the EDF is composed of GCS dissolved in a mixture of WFI containing PRG, surfactants (SR polysorbate 20, SR P35 Castor oil), and cosurfactant (SR PEG400), where each excipient has its own specific role. This outer aqueous shell plays a crucial role in the pharmacological effect of the prepared EDF. This shell is responsible for the EDF bioadhesion, rapid onset of the drug effect, long precorneal residency, penetration enhancement, and the demulcent effect. The bioadhesion and the long precorneal residency are due to GCS, while the penetration enhancement property is due to the presence of surfactants, in addition to the demulcent effect due to the SR PEG400, which is a common ingredient of marketed artificial tears [42,43]. Furthermore, the outer aqueous shell enhanced the mixing of the EDF with the tear film, allowing good spreading of the drug over the corneal surface and formation of a homogeneous transparent thin film of the EDF that resulted in a more efficient prolonged contact time of the drug with the corneal epithelium.

### 3.2. Exploratory In Vivo Studies

#### 3.2.1. Drop Volume Response Study

**Figure 2:**
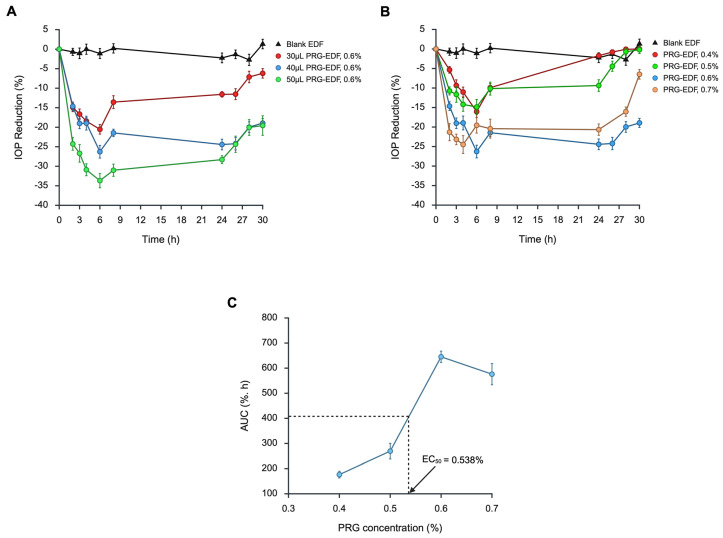
(**A**) Percentage IOP reduction vs. time profiles of DB rabbits (*n* = 6 rabbits, 12 eyes) after topical application of different volumes of PRG-EDF, 0.6% eye drops. (**B**) Percentage IOP reduction vs. time profiles of DB rabbits (*n* = 6 rabbits, 12 eyes) after topical application of PRG-EDF eye drops containing different concentrations of PRG from 0.4 to 0.7% *w*/*w*. (**C**) AUC (%.h) versus PRG concentrations plot. The calculated EC_50_ of PRG is 0.538% *w*/*w*. Created in BioRender. Maria, D. (2026) https://BioRender.com/7o4nnqv.

This study evaluated the effect of the dose volume on the IOP-lowering efficacy of PRG-EDF, 0.6%. For this purpose, six DB rabbits were dosed bilaterally with three dose volumes, including 30, 40, or 50 μL. Figure 2A illustrates % IOP versus time after administration of the various drop volumes, and Table 2 lists a summary of the PD parameters. The data demonstrated that there was a significant dose-volume dependent response in the IOP-lowering efficacy of PRG-EDF, including both % IOP reduction (overall *p* < 0.0001) and AUC (overall *p* < 0.0001) in the following order: 50 µL > 40 µL > 30 µL. In case of traditional eye drops, we do not expect to measure any significant difference in the response between different dose volumes because the eye can hold only 10–20 µL of the aqueous solution, and any amount beyond this will either drain through the nasolacrimal duct or overflow the eyelid [44]. In contrast, the eye can hold a larger volume of PRG-EDF because of its bioadhesiveness due to the presence of the bioadhesive polymer. In addition, the presence of surfactants/cosurfactants can facilitate the spreading of the EDF evenly over the ocular surface to form a thin and homogeneous film because of their ability to decrease the interfacial tension with the eye surface [15,16,17,18,19,20,21,22,23,24,25,26,27,28,29,30,31,32,33,34,35,36,37,38,39,40,41,42,43,44,45]. Although there was a significant difference between 40 µL and 50 μL dose volumes when evaluating the % IOP reduction and AUC, 40 μL was selected. Because the eye drop containers on the market typically deliver 30 or 40 μL doses, we selected 40 µL. Unfortunately, no eye drop container on the market can deliver a 50 μL dose. Thus, 40 μL is a more common drop size used by standard container closure systems. Impressively, 40 µL demonstrated a reasonable IOP-lowering efficacy with a rapid onset (T_max_ approximately 6 h) and long duration for more than 30 h.

#### 3.2.2. Dose Response and EC_50_ Determination Study

This study evaluated the dose response of PRG-EDF at four dose concentration levels—0.4, 0.5, 0.6, and 0.7% *w*/*w*—to determine the minimal concentration of PRG that is required to produce the maximal reduction in IOP. Figure 2B demonstrates the % IOP reduction versus time profiles after topical application of a single 40 µL drop of PRG-EDF containing various concentrations of PRG or the blank EDF (*n* = 6 rabbits, 12 eyes per condition), and Table 3 lists the summary of the calculated PD parameters. These data demonstrate that PRG-EDF eye drops containing different PRG concentrations were able to reduce the IOP of DB rabbits, and this reduction is directly related to the concentration of PRG. Table 3 reveals that the highest IOP reduction response was achieved with both 0.6% and 0.7% dose levels, with no significant difference in the % IOP reduction (*p* > 0.9999), the duration of action (T_end_, *p* > 0.9999), and the total area under the % IOP reduction versus time curve (*p* = 0.6500) (Table 4). This non-significance in the drug response between 0.6% and 0.7% dose levels may be attributed to the saturation at the target site at the 0.6% dose level; therefore, the addition of more drug has no further effect on the pharmacological response. Comparison of PD parameters of the 0.5% and 0.6% dose levels demonstrates that there was a significant difference in % IOP reduction (*p* = 0.0007), T_end_ (*p* = 0.0044), and the total area under the % IOP reduction versus time curve (*p* < 0.0001). Thus, the 0.6% dose level can be considered the optimal PRG level in our EDF because it is the lowest concentration that produced the maximum IOP-lowering effect. These data demonstrate that our new PRG-EDF is equally effective as our prototype PRG-ME formulation.

The EC_50_ was calculated from the graph resulting from plotting the AUC versus PRG concentration and then extrapolating to the concentration that produced 50% of the maximum AUC value (Figure 2C). AUC data rather than the % IOP reduction data were used because the AUC encompasses both the amplitude of IOP reduction and duration of action, compared with the % IOP reduction, which only reflects the amplitude of IOP reduction. Thus, AUC is considered the best representation of PRG IOP-lowering efficacy. The data revealed that the EC_50_ for IOP-lowering of PRG-EDF is 0.538% *w*/*w*. In conclusion, these data demonstrate a concentration-related response in the IOP-lowering efficacy of PRG with early onset (T_max_ approximately 6 h) and extended duration of action (T_end_ > 30 h) and optimal response at a 0.6% *w*/*w* dose level that produced 27.6 ± 1.2% IOP reduction.

### 3.3. In Vitro Characterizations of PRG-EDF

#### 3.3.1. pH, Average Droplet Size, Polydispersity Index (PDI), and Zeta Potential (ZP)

Because of the tears natural buffering capacity, the eye can tolerate ophthalmic formulations with a wide pH range (from pH 3.5 to 8.5). pH values of the prepared EDF eye drops, either the blank or the medicated, were near the physiological range (Table 5). Thus, PRG-EDF eye drops should be tolerated by the eye without any irritation or discomfort [46]. Table 5 lists the mean droplet size, PDI, and ZP values of both blank and medicated EDF. The droplet size of the prepared blank or medicated EDF is <30 nm with PDI values < 0.26. This tiny droplet size is essential to ensure good efficacy as it greatly facilitates the transcorneal penetration of the drug. A droplet size less than 200 nm is required for successful passive drug transport through biological membranes [14,15,16,17,18,19,20,21,22,23,24,25,26,27,28,29,30,31,32,33,34,35,36,37,38,39,40,41,42,43,44,45,46,47]. In addition, the small PDI values indicate that our prepared EDF is homogeneous and has a narrow droplet size distribution [48]. This small droplet size and size distribution may be attributed to the use of high concentrations of surfactants along with the use of PEG400 as a cosurfactant. Hence, the surfactant/cosurfactant mixture decreased the interfacial tension and improved the emulsification efficiency [49]. Concerning the zeta potential, both blank and medicated EDF have high negative surface charges. It has been reported that the high surface charge of the droplet is a very important stability factor because it creates a continuous motion due to repulsion between the droplets and prevents their agglomeration. This high negative zeta potential may be due to the use of soybean lecithin as an anionic surfactant [50,51].

#### 3.3.2. Determination of Viscosity

**Figure 3:**
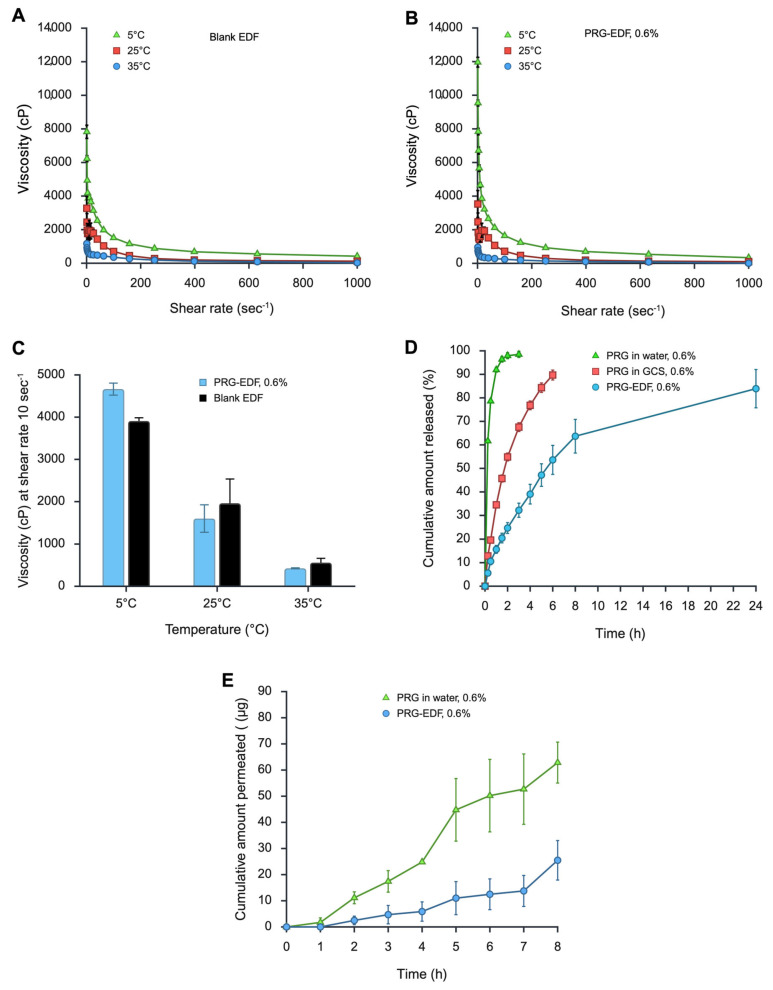
Flow behavior profiles of (**A**) blank EDF and (**B**) PRG-EDF, 0.6%, at three temperatures, 5 °C, 25 °C, and 35 °C (mean ± SEM; *n* = 3); the rheograms illustrated that viscosity decreased with increasing shear rate (shear-thinning). (**C**) Viscosity histograms of blank EDF and PRG-EDF, 0.6%, at a shear rate of 10 s^−1^ at the three temperatures, 5 °C, 25 °C, and 35 °C. Viscosity decreased with increasing temperature. (**D**) Cumulative amount of PRG released (%) from PRG-EDF, 0.6% eye drops, and controls (mean ± SEM; *n* = 3). PRG-EDF, 0.6%, succeeded in slowing and sustaining the release of PRG up to 24 h, in contrast to the control formulations that exhibited rapid release of the entire drug in the first few hours. (**E**) Ex vivo transcorneal permeability profiles of PRG from the PRG-EDF, 0.6% eye drops, and PRG aqueous solution, 0.6%, as a control formulation (mean ± SEM, *n* = 4). PRG-EDF, 0.6%, demonstrated a controlled transcorneal penetration compared to PRG aqueous solution. Created in BioRender. Maria, D. (2026) https://BioRender.com/2eonmx6.

Table 6 lists the measured viscosity values of blank EDF and PRG-EDF, 0.6% eye drops. The viscosity was measured at a shear rate range from 1 to 1000 S^−1^, to be related to the physiologically relevant blinking conditions. Because human blinking can generate transient shear rates higher than 100 s^−1^, the rheological measurements were determined at a shear rate range up to 1000 s^−1^ to mimic in vivo behavior [52]. Figure 3A,B demonstrate that the viscosity of both blank and medicated EDF decreases when the shear rate increases at the three temperatures used, 5 °C, 25 °C, and 35 °C, indicating their non-Newtonian pseudoplastic shear-thinning flow behavior. One of the reasons for this flow behavior is that the GCS, as a long-chain polymer, could contribute to the shear-thinning behavior of the formulation. At rest (low shear rate), the polymer chain coils and entangles to form a network that entraps the liquid ME in its lattice structure. Upon increasing the shear rate, the end-to-end distance of the polymer chain increases and the polymer chain tends to disentangle and align with the flow direction, which releases the entrapped fluid, resulting in a drastic decrease in the formulation viscosity [53]. This rheological behavior is ideal for topical ocular formulations because it allows the formulations to be more viscous at rest (low shear) and less viscous during blinking (high shear) [52]. Being viscous at rest helps the formulation to remain on the eye surface and prevent its drainage, while being less viscous during blinking allows for normal blinking movement and minimizes patient discomfort [54,55].

To compare the effect of different temperatures on the viscosities of EDF, Figure 3C illustrates the viscosity of both blank and medicated EDF at a fixed shear rate (10 s^−1^) at different temperatures. The data demonstrate that viscosity decreased with increasing temperature from 5 °C to 35 °C, which would lead to a reduction in the viscosity of the formulation on the eye surface (35 °C), thus limiting interference with the normal blinking movement. Comparison of the blank and medicated EDFs revealed no significant difference between their viscosities at a fixed shear rate (10 s^−1^) at the three used temperatures, 5 °C, 25 °C, and 35 °C (*p* = 0.5978, *p* > 0.9460, and *p* = 0.9994, respectively) (Figure 3C).

#### 3.3.3. In Vitro Drug Release

For the drug to be absorbed and achieve its pharmacological effect, it must first be released from its carrier; thus, the drug in vitro release behavior from a specific formulation is a determinant of its pharmacological effect after administration. Figure 3D demonstrates the in vitro release profiles of PRG from PRG-EDF, 0.6%, as well as two control formulations (PRG in water, 0.6%, and PRG in GCS, 0.6%). Comparing the rate of PRG release from the PRG-EDF and the two controls yields the following order: PRG in water > PRG in GCS > PRG-EDF. After 1 h, the cumulative percentage of PRG released from the PRG aqueous solution, PRG in GCS, and PRG-EDF was 91.9 ± 1.0%, 34.5 ± 1.3%, and 15.6 ± 1.7%, respectively. The rapid release of PRG from the aqueous solution can be attributed to its free water solubility, which makes it immediately available for release. Hence, the rate-limiting step here is the diffusion through the semipermeable regenerated cellulose membrane. However, in the case of PRG in GCS, PRG diffuses first through the viscous polymeric solution before passing through the semipermeable regenerated cellulose membrane. Thus, this additional diffusion step retarded PRG release from GCS compared to aqueous solution. In contrast, PRG-EDF, 0.6%, demonstrated an initial fast release followed by a sustained release for up to 24 h. This initial fast release of PRG is likely due to the PRG (70%) that was incorporated into the aqueous polymeric bioadhesive shell, which released upon contact with the release medium after simple diffusion through the polymeric bioadhesive aqueous shell. However, for PRG (30%) that was incorporated in the inner aqueous core to be released from PRG-EDF, it must pass through several layers before reaching the semipermeable regenerated cellulose membrane, including the movement from the innermost aqueous core through the oil layer barrier, followed by diffusion through the viscous GCS layer in the outer aqueous shell. These steps greatly retarded PRG release from PRG-EDF, which succeeded in sustaining the release of PRG for up to 24 h compared to the control formulations that exhibited a rapid PRG release for 3–6 h. Another reason for the sustained release may be the possibility of electrostatic interaction or hydrogen bonding between PRG zwitterion and phosphotidyl choline zwitterion in soyabean lecithin, which sustains the drug release from the aqueous core [56].

Table 7 presents the kinetic analysis of PRG in vitro release data from PRG-EDF, 0.6%, and the two controls, wherein the results represent the correlation coefficient values calculated by fitting the release data to different release kinetic models, including zero, first, and Higuchi. After screening of the release data against various release kinetic models, the results demonstrated that the release kinetics of PRG from PRG EDF, 0.6% eye drops, is a biphasic process wherein the two phases follow different release kinetic models. The first release phase (0.25–5 h) obeys zero order kinetics, while the second phase (started after 5 h) follows the Higuchi model. This biphasic release behavior of PRG from PRG-ED, 0.6% eye drops, may be due to the presence of PRG in the two aqueous systems of the EDF (core and shell aqueous systems), which are separated by the oil shell. Therefore, the presence of PRG in the aqueous bioadhesive shell of the EDF could be the reason for the first release phase (zero order kinetics). This is because the PRG in the aqueous bioadhesive shell is ready for direct release by simple diffusion through the bioadhesive polymeric aqueous shell. In the first release phase, the PRG amount released is immediately renewed by the PRG in the aqueous core system, which acts as a reservoir for PRG. Subsequently, PRG concentration in the aqueous bioadhesive shell remains nearly constant, and the release rate is independent of the drug concentration (i.e., zero order kinetics). However, at the second release phase, the release kinetics are converted to another model, the Higuchi diffusion mechanism, after all PRG in the aqueous core system is released, and thus the drug release rate depends on the PRG concentration in the aqueous bioadhesive shell [15]. In addition, the release kinetic data from the two controls followed a Higuchi model (the model with the highest correlation coefficient) [15,16,17,18,19,20,21,22,23,24,25,26,27,28,29,30,31,32,33,34,35,36,37,38,39,40,41,42,43,44,45,46,47,48,49,50,51,52,53,54,55,56,57]. Further analysis of the release data using the Korsmeyer–Peppas model revealed that the values of Korsmeyer–Peppas release exponents (*n*) of PRG in PRG-EDF, 0.6%, were <0.5 (second phase), those of PRG in GCS, 0.6%, were >0.5 and <1.0, and those of PRG in water, 0.6%, were <0.5. These results suggested that the mechanism of PRG release from both PRG-EDF and PRG in water was a Fickian diffusion mechanism or a pure diffusion mechanism. In contrast, the release mechanism of PRG from PRG in GCS was a non-Fickian diffusion mechanism or anomalous diffusion mechanism (i.e., a mixture of diffusion and polymer swelling and/or erosion) [58] (Table 7).

### 3.4. Ex Vivo Transcorneal Permeability

To evaluate the transcorneal permeability of PRG from the prepared PRG-EDF, 0.6%, as well as from the control formulation, PRG in water, 0.6%, we used fresh rabbit corneas excised from the whole eyes of New Zealand white rabbits. To maintain corneal viability throughout the 8 h experiment (Figure 3E), we used BSS-Plus irrigating solution as a receptor medium. As previously mentioned, PRG is a BCS class I drug, which has high aqueous solubility and high permeability. The results demonstrate that PRG-EDF, 0.6%, can maintain the inherent high permeability of PRG with no significant difference in the permeation rates (*dM/dt*), flux values (*J*), or permeability coefficients (*P*) compared to the control formulation (*p* = 0.996) (Table 8). However, we expect that, in an in vivo study, the PRG-EDF will demonstrate superior efficacy than the control formulation due to its bioadhesiveness, which can maintain PRG on the ocular surface for an extended period of time. In contrast, the control, PRG in water, 0.6%, is expected to drain out of the eye within the first 5 min post dosing in the in vivo study.

### 3.5. Pregabalin-EDF In Vivo Evaluations

#### 3.5.1. Comparison of the IOP-Lowering Efficacy of PRG-EDF, 0.6%, with Marketed Glaucoma Medications

**Figure 4:**
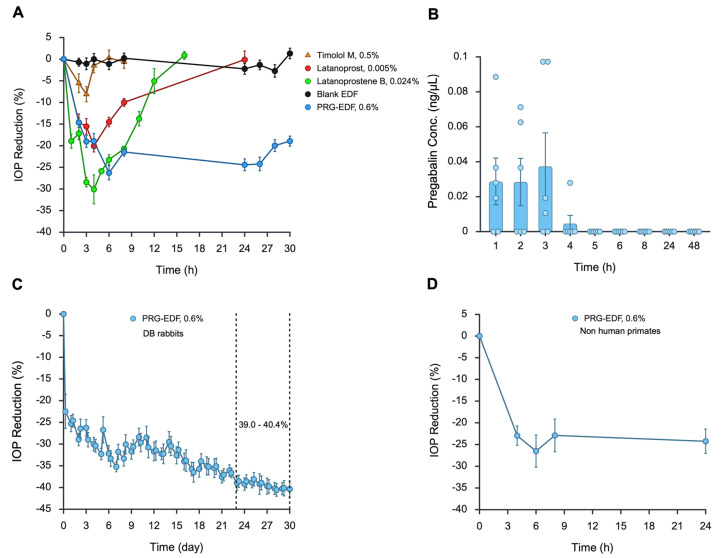
(**A**) Percentage IOP reduction vs. time profiles after topical application of a single 40 μL dose of PRG-EDF, 0.6%, compared to commercial glaucoma medications. (**B**) PRG plasma levels after a single topical ocular bilateral application of 40 μL of PRG-EDF, 0.6%, in DB rabbits (mean ± SEM; *n* = 6). (**C**) Percentage IOP reduction vs. time profiles of DB rabbits after repeated daily bilateral topical application of 40 μL of PRG-EDF, 0.6%, for a month (mean ± SEM; *n* = 6 rabbits, 12 eyes). Repeated daily dosing with PRG-EDF, 0.6%, eye drops for 30 days, did not result in a decrease in efficacy of the formulation, and IOP fluctuations were eliminated. Importantly, IOP decreased with daily dosing until a plateau was reached. (**D**) Percentage IOP reduction vs. time profile after topical application of a single dose of PRG-EDF, 0.6%, in non-human primates (mean ± SEM; *n* = 7 baboons, 14 eyes). Created in BioRender. Maria, D. (2026) https://BioRender.com/cog8la3.

This study compared the IOP-lowering efficacy of PRG-EDF, 0.6% eye drops to that of several commercial glaucoma eye drops, specifically timolol maleate 0.5%, latanoprost 0.005%, and latanoprostene bunod 0.024%. A single dose design was selected for the study, in which each formulation was topically applied to DB rabbits (*n* = 6 rabbits, 12 eyes). Four groups of DB rabbits (six rabbits per group) were dosed bilaterally with 40 μL of each formulation. Figure 4A presents the % IOP reduction versus time profiles after topical application of a single dose of the tested formulations. Table 9 lists the summary of the calculated PD parameters, and Table 10 illustrates their statistical analysis. The results demonstrate that, among all tested commercial glaucoma medications, latanoprostene bunod provided the maximum IOP-lowering effect, with a reduction in IOP of 30.9 ± 2.5% that returned to baseline at 14.7 ± 1.3 h and AUC of 241.4 ± 14.6%.h. Comparing PRG-EDF with latanoprostene bunod (the commercial formulation with the largest amplitude of IOP reduction) revealed the superiority of PRG-EDF in its overall IOP-lowering efficacy. Although there is no significant difference in the % IOP reduction between them (*p* = 0.5598), PRG-EDF possesses a longer duration of action (T_end_ > 30 h, *p* < 0.0001) and AUC of 645.2 ± 22.5%.h, which is almost three times that of latanoprostene bunod (241.4 ± 14.6%.h, *p* < 0.0001). This superiority in EDF efficacy is likely due to its specific engineering with PRG distributed in both the aqueous core and the aqueous shell. PRG in the aqueous core (30% of the drug load) is responsible for the longer duration of action, while PRG in the aqueous shell (70% of the drug load) contributes to the rapid onset of action. Furthermore, the main factors that led to the superiority of PRG-EDF are its bioadhesiveness, due to the presence of GCS [34], and its corneal penetration enhancement ability, due to the use of surfactant/cosurfactant mixture [42,43]. Collectively, from the presented data, the tested formulations can be arranged in the following order according to the superiority of their IOP-lowering potency: PRG-EDF > latanoprostene bunod > latanoprost > timolol maleate.

#### 3.5.2. Exploratory Plasma Pharmacokinetic (PK) Study After Topical Ocular Administration of a Single Dose of PRG-EDF, 0.6%

The purpose of this study is to determine possible PRG systemic absorption after a single topical dose of PRG-EDF, 0.6%. Because glaucoma is a lifelong disease, it is preferable to limit systemic absorption after topical application to avoid any possible systemic side effects. Plasma PRG levels were determined using a sensitive liquid chromatography tandem mass spectroscopic method that has a 2.5 pg/µL LOQ and 0.76 pg/µL LOD, which facilitates the detection of any traces of PRG in the plasma [14,15,16,17]. The data in Figure 4B illustrate that trace levels of PRG were detected in plasma during the first 4 h after dosing, with a maximum value of 0.037 ± 0.019 µg/mL after 3 h. After 4 h, PRG plasma levels were below the detection limit of the quantification method. Although there was some PRG detected in the systemic circulation after topical application of PRG-EDF, it is at a very low and safe level compared to that achieved after oral PRG administration. It is reported that PRG plasma level can reach 3.78 µg/mL after a single oral dose of 150–600 mg of PRG in humans [59] and 10.1 µg/mL after a single oral dose of 5 mg/kg in cats [60]. Thus, the detected PRG plasma levels were orders of magnitude lower than those measured after oral administration at clinical dosing levels, which strongly suggests that topical ocular dosing with PRG-EDF, 0.6%, will have no or negligible systemic side effects. This low systemic absorption is likely due to the localization of the formulation on the eye surface due to its bioadhesion [34].

#### 3.5.3. Long-Term Efficacy Study: 30 Days of a Single Daily Application of PRG-EDF, 0.6% in DB Rabbits

Loss or decreased IOP-lowering effect upon prolonged use can be a common drawback of some glaucoma medications, such as timolol maleate [61]. For this reason, we tested the change in the IOP-lowering effect of PRG-EDF, 0.6%, during a month of daily topical administration to DB rabbits. Figure 4C presents the % IOP reduction profile of the DB rabbits over 30 days. During the study, IOP was measured at baseline on the first day, followed by twice-daily measurements: at T_max_, which is 6 h (determined from previous studies), and after 24 h from the previous dose. The data illustrated in Figure 4C demonstrate that at the T_max_ of the first day, PRG-EDF induced a rapid initial % IOP reduction of 22.5 ± 3.9%. By continuing the daily application of the PRG-EDF, a gradual increase in the IOP-lowering effect was observed that reached its maximum at ~day 28. Interestingly, the % IOP reduction was 39.0–40.4% after 23 days of single daily dosing, which is significantly higher than the reduction observed during the first day (*p* < 0.0001). The reason behind the gradual increase in the PRG-EDF pharmacological response is unknown but may be attributed to the possible accumulation of the drug at the receptor site due to the daily dosing or structural changes in the drainage pathway in the trabecular meshwork, which reached its maximum accumulation after 28 days. Impressively, after 28 days of once daily application, the IOP values remained in the physiological range, which protects the eyes from ocular hypotony that could happen if the IOP falls below 10 mmHg [22,23,24,25,26,27,28,29,30,31,32,33,34,35,36,37,38,39,40,41,42,43,44,45,46,47,48,49,50,51,52,53,54,55,56,57,58,59,60,61,62]. In conclusion, the results demonstrate that PRG-EDF, 0.6% eye drops, can maintain its IOP-lowering ability with a single daily dose of 40 μL without a decrease in drug pharmacological effect upon prolonged use.

#### 3.5.4. IOP-Lowering Efficacy of PRG-EDF, 0.6% in Non-Human Primates After a Single Topical Application

Baboons were selected as a second species to confirm the IOP-lowering efficacy of PRG-EDF, 0.6%, and to bridge the gaps between the rabbit data and humans because of their very close phylogenetic relationship to humans, and also because they share many genetic, biochemical, physiologic, and anatomic characteristics [63]. In addition, NHP eyes are structurally and functionally very similar to human eyes, which makes them an ideal animal model to test new eye drops before being used on humans [64]. Seven baboons were used in a single dose study design, in which each animal received a 40 µL drop in both eyes. Figure 4D presents the % IOP reduction versus time profile after topical application of PRG-EDF, 0.6% eye drops, in baboons. The data demonstrate that baboons experienced a good response to the IOP-lowering effect of PRG-EDF eye drops. They demonstrated a % IOP reduction of 26.5 ± 3.7% after 6.0 ± 0.5 h post dosing that did not return to baseline after 24 h from application, with a total area under the % IOP reduction versus time curve of 528.9 ± 62.1%.h. These positive results using NHP can be considered a promising bridge to future clinical trials in humans [65].

#### 3.5.5. Bioadhesion Study of PRG-EDF on the Corneal Surface of DB Rabbits

**Figure 5:**
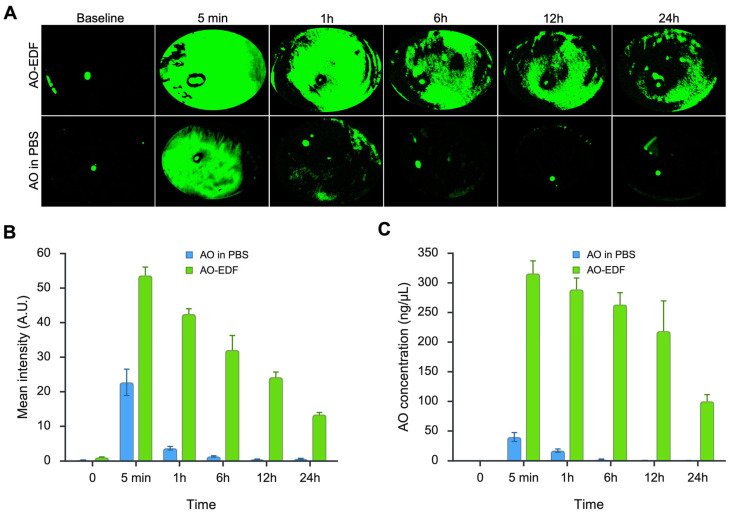
Bioadhesion assessment of AO-EDF and AO in PBS in DB rabbits (mean ± SEM; *n* = 5). (**A**) Fluorescence images of the DB rabbits eye surface at different time intervals after a single application of either AO-EDF or AO in PBS. EDF remains on the eye surface for up to 24 h. However, AO in PBS is drained from the eye surface within the first hour after application. (**B**) Quantitative measurement of the fluorescence intensity from the rabbit eye photos evaluated using ImageJ software. The graph demonstrates that EDF can maintain a high level of AO on the eye surface for up to 24 h. (**C**) The measured concentration of AO in tears of DB rabbits (mean ± SEM; *n* = 5) dosed with a single drop of either AO-EDF or AO in PBS. Created in BioRender. Maria, D. (2026) https://BioRender.com/mzv6vmq.

An in vivo bioadhesion study was conducted to evaluate the formulation residency time on the eye surface and the ability of the formulation to adhere to the corneal surface, where the formulation can act as a drug reservoir that continuously releases PRG to extend the duration of its IOP-lowering effect. For that purpose, EDF-containing acridine orange, 0.6% (AO-EDF), a fluorescent cargo, was topically applied to the DB rabbit eyes. As is evident in the images of the DB rabbit eye surfaces presented in Figure 5A, AO-EDF remains on the eye surface for at least 24 h after a single dose. In contrast, the fluorescent dye, when dosed in PBS (AO in PBS), nearly disappeared from the control eye within one hour. Quantification of the fluorescent intensity from the rabbit eye photos using ImageJ illustrated that significant levels of the EDF remained on the eye surface for up to 24 h (Figure 5B). Fluorescent intensities, calculated as percentages from the initial value that was measured after 5 min, were 80, 60, 45, and 25% after 1, 6, 12, and 24 h, respectively. However, the fluorescent intensity quantification of the eyes that received AO in PBS demonstrated trace amounts of the dye over all time points after one hour (Figure 5B). Figure 5C demonstrates the results of the quantitative determination of acridine orange concentrations in tears. These data illustrate that the fluorescent dye was measured in tears in a very high concentration for up to 24 h post application of AO-EDF compared to the tear samples from the control eye dosed with AO in PBS, which revealed traces of the dye in tears (Figure 5C). The high fluorescence levels expressed by the eyes treated with AO-EDF in comparison with the eyes treated with AO in PBS are likely due to the ability of the EDF to remain on the eye surface for a period of time that exceeded 24 h due to the presence of GCS bioadhesive polymer in the outer aqueous shell of the EDF system. These data can explain the long duration of the IOP-lowering efficacy of PRG-EDF observed in various in vivo models.

## 4. Conclusions

In the current study, we present PRG-EDF, which is a promising glaucoma medication that incorporates a new class of glaucoma therapeutic agents. PRG-EDF was carefully engineered and evaluated to reach a state of readiness for clinical evaluation. PRG-EDF was manufactured exclusively using excipients with proven safety profiles, and the produced formulation was extensively evaluated using various testing methods. Evaluation of several drop volumes of PRG-EDF, 0.6%, revealed that 40 μL produced a reasonable IOP reduction within the acceptable range of drop volumes used in marketed eye drops. Evaluation of the IOP-lowering response of PRG-EDF containing several concentrations of PRG illustrated that 0.6% PRG is the lowest concentration that produces a maximum IOP-lowering effect with an EC_50_ value of 0.538% *w*/*w*. In vitro evaluations revealed that PRG-EDF, 0.6%, provided extended release of PRG for up to 24 h compared to the controls, which released 100% of the drug within the first few hours. Droplet size analysis demonstrated that PRG-EDF has a minuscule droplet size (<30 nm), along with a narrow size distribution, as indicated by the small PDI values. Also, PRG-EDF has a high negative surface charge, which suggests a long shelf-life stability. To evaluate the position of our PRG-EDF on a plausible market map, the efficacy of PRG-EDF, 0.6% eye drops, was compared with three of the most used marketed glaucoma medications. Impressively, the comparison revealed the superiority of our formulation, as illustrated by higher IOP-lowering ability and prolonged duration of action. Regarding the systemic safety profile of PRG-EDF, PRG was only detected in plasma as traces in the first few hours post-dosing and completely disappeared 4 h after application. Long-term daily dosing proved that PRG-EDF can maintain its IOP-lowering ability without any diminishment or loss of potency, which is very common for some glaucoma medications. In contrast, PRG-EDF potency was increased with time until reaching its maximum within 4 weeks. Interestingly, the in vivo bioadhesion demonstrated that EDF can remain on the ocular surface with a measurable amount of the fluorescent dye in tears for up to 24 h compared to the dye aqueous solution, which almost disappeared within the first hour post-application. Collectively, we conclude that PRG-EDF, 0.6%, is a highly promising once-daily glaucoma medication that is suitable for clinical evaluation.

## Figures and Tables

**Table 1:** Composition of PRG-EDF, 0.6% *.

Ingredient	%*w*/*w*	Category
Pregabalin	0.60 *	FDA-approved
Crodamol GTCC	7.70	FDA-approved
Super refined brij O2	3.90	FDA-approved
Span 83	3.90	FDA-approved
Soybean L-α-Lecithin	5.10	FDA-approved
Super refined polysorbate 20	7.30	FDA-approved
Super refined P35 castor oil	7.30	FDA-approved
Super refined PEG400	22.20	FDA-approved
Glycol Chitosan	0.02	GRAS
WFI	41.98	FDA-approved

* Four concentrations of PRG were prepared: 0.4, 0.5, 0.6, and 0.7% *w*/*w*.

**Table 2:** Pharmacodynamic parameters after bilateral topical application of 30, 40, or 50 μL of PRG ^a^ EDF ^b^, 0.6% to DB rabbits (*n* = 6 rabbits, 12 eyes).

Pharmacodynamic Parameters (Mean ± SEM)	Dose Volume of PRG-EDF, 0.6%		
	30 μL	40 μL	50 μL
Baseline IOP ^c^ (mmHg)	20.1 ± 0.2	20.0 ± 0.1	20.6 ± 0.6
IOP at T_max_^d^ (mmHg)	15.7 ± 0.2	14.5 ± 0.2	13.4 ± 0.2
∆IOP	−4.4 ± 0.2	−5.7 ± 0.4	−7.2 ± 0.5
T_max_ (h)	5.01 ± 0.5	5.75 ± 0.4	5.33 ± 0.5
% IOP reduction at T_max_	22.1 ± 0.7	27.6 ± 1.2	34.5 ± 1.7
T_end_^e^ (h)	>30	>30	>30
AUC ^f^ (%.h)	377.6 ± 24.4	645.2 ± 22.5	818.9 ± 37.8

^a^ PRG: pregabalin. ^b^ EDF: enhanced delivery formulation. ^c^ IOP: intraocular pressure. ^d^ T_max_: time to maximum response in hours. ^e^ T_end_: time to end of response in hours. ^f^ AUC (%.h): total area under % IOP reduction versus time curve.

**Table 3:** Pharmacodynamic parameters after topical application of a 40 µL single dose of PRG-EDF containing concentrations of PRG from 0.4 to 0.7% *w*/*w* to DB rabbits (mean ± SEM; *n* = 6 rabbits, 12 eyes).

Pharmacodynamic Parameters	Concentration of PRG ^a^ in EDF ^b^ Eye Drops (% *w*/*w*)			
	0.4%	0.5%	0.6%	0.7%
Baseline IOP ^c^ (mmHg)	20.0 ± 0.2	19.8 ± 0.2	20.0 ± 0.1	20.2 ± 0.4
IOP at T_max_^d^ (mmHg)	15.8 ± 0.8	16.0 ± 0.3	14.5 ± 0.2	14.6 ± 0.3
∆IOP(mmHg)	−3.4 ± 0.3	−3.8 ± 0.3	−5.7 ± 0.4	−5.6 ± 0.4
T_max_ (h)	4.8 ± 0.5	4.7 ± 0.4	5.75 ± 0.4	3.5 ± 0.5
% IOP reduction at T_max_	16.9 ± 1.4	19.1 ± 1.5	27.6 ± 1.2	27.5 ± 1.6
T_end_^e^ (h)	27.0 ± 0.6	28.2 ± 0.5	>30	>30
AUC ^f^ (%.h)	176.5 ± 13.3	269.3 ± 31.1	645.2 ± 22.5	596.9 ± 42.1

^a^ PRG: pregabalin. ^b^ EDF: enhanced delivery formulation. ^c^ IOP: intraocular pressure. ^d^ T_max_: time to maximum response in hours. ^e^ T_end_: time to end of response in hours. ^f^ AUC (%.h): total area under % IOP reduction versus time curve.

**Table 4:** Statistical comparisons of PD parameters after topical application of PRG ^a^ EDF ^b^ containing concentrations of PRG from 0.4 to 0.7% *w*/*w* to DB rabbits.

Pharmacodynamic Parameters	% IOP ^c^ Reduction at T_max_	T_max_^d^	T_end_^e^	AUC ^f^
Overall *p* value ^g^	<0.0001	0.0088	<0.0001	<0.0001
0.4% vs. 0.5%	0.7069 ^h^	0.9991	0.5634	0.1271
0.4% vs. 0.6%	<0.0001	0.3850	<0.0001	<0.0001
0.4% vs. 0.7%	<0.0001	0.2001	<0.0001	<0.0001
0.5% vs. 0.6%	0.0007	0.3151	0.0044	<0.0001
0.5% vs. 0.7%	0.0007	0.2533	0.0044	<0.0001
0.6% vs. 0.7%	>0.9999	0.0041	>0.9999	0.6500

^a^ PRG: pregabalin. ^b^ EDF: enhanced delivery formulation. ^c^ IOP: intraocular pressure. ^d^ T_max_: time to maximum response in hours. ^e^ T_end_: time to end of response in hours. ^f^ AUC (%.h): total area under % IOP reduction versus time curve. ^g^ Overall *p*-value represents the outcome of the one-way ANOVA analysis. ^h^ Individual *p*-values represent the outcome of Tukey’s multiple comparisons test.

**Table 5:** pH, Droplet Size, Zeta Potential, and PDI of blank EDF and PRG-EDF, 0.6%.

EDF	pH	Droplet Size (nm)	PDI	Zeta Potential (mV)
Blank	7.34 ± 0.02	29.81 ± 0.23	0.258 ± 0.00	−56.97 ± 0.99
Medicated	7.18 ± 0.04	29.03 ± 0.45	0.258 ± 0.01	−26.60± 1.09

**Table 6:** Viscosity (cP) of blank EDF and PRG-EDF, 0.6% eye drops at different shear rates and three temperatures.

Temperature	Shear Rate (S^−1^)	EDF Viscosity (cP)	
		Blank	Medicated
5 °C	1	7826.2 ± 391.2	11,962.6 ± 290.9
	10	3909.3 ± 75.9	4663.2 ± 142.7
	100	1513.3 ± 12.0	1645.4 ± 32.1
	1000	417.4 ± 11.5	338.9 ± 67.4
25 °C	1	3271.5 ± 697.4	3531.9 ± 802.3
	10	1959.2 ± 576.4	1598.9 ± 324.9
	100	699.5 ± 17.1	718.5 ± 45.3
	1000	121.3 ± 48.9	102.7 ± 35.9
35 °C	1	1165.7 ± 116.8	960.8 ± 16.6
	10	559.7 ± 103.1	426.1 ± 11.3
	100	354.9 ± 40.4	242.7 ± 9.1
	1000	25.8 ± 21.1	6.6 ± 0.2

**Table 7:** Kinetics analysis of PRG in vitro release from PRG-EDF, 0.6%, PRG in water, 0.6%, and PRG in GCS, 0.6%.

Formulation	Coefficient of Determination (R^2^)				Korsmeyer–Peppas		Drug Transport Mechanism	Release Mechanism
	Zero		First	Higuchi	r^2^	*n*		
PRG in water	0.513 ± 0.006		0.618 ± 0.004	0.795 ± 0.005	0.896 ± 0.003	0.190 ± 0.006	Fickian	Pure diffusion
PRG in GCS	0.916 ± 0.003		0.768 ± 0.010	0.991 ± 0.001	0.988 ± 0.003	0.627 ± 0.015	Non-Fickian	Anomalous diffusion
PRG-EDF	1st phase	0.979 ± 0.002	0.841 ± 0.018	0.978 ± 0.000	----	----	----	Diffusion
	2nd phase	0.948 ± 0.006	0.919 ± 0.007	0.965 ± 0.005	0.964 ± 0.005	0.306 ± 0.014	Fickian	

**Table 8:** Ex vivo transcorneal permeability parameters of PRG from PRG-EDF, 0.6% and PRG in water, 0.6%.

Formulation	Rate of Permeation (*dM*/*dt*) (μg·min^−1^)	Flux (μg·cm^−2^·min^−1^)	Permeability Coefficient (*P*) × 10^5^ (cm·min^−1^)
PRG-EDF	0.095 ± 0.05	0.149 ± 0.08	2.49 ± 1.28
PRG in water	0.095 ± 0.03	0.149 ± 0.05	2.48 ± 0.76

**Table 9:** Pharmacodynamic parameters after topical application of PRG ^a^-EDF ^b^, 0.6%, and marketed glaucoma medications to DB rabbits ^c^.

Pharmacodynamic Parameters	Tested Eye Drops			
	Timolol Maleate 0.5%	Latanoprost 0.005%	Latanoprostene Bunod 0.024%	PRG-EDF 0.6%
Baseline IOP ^d^ (mmHg)	19.8 ± 0.7	18.3 ± 0.2	19.3 ± 0.3	20.0 ± 0.1
IOP at T_max_^e^ (mmHg)	18.2 ± 0.3	14.7 ± 0.4	13.3 ± 0.3	14.5 ± 0.2
∆IOP (mmHg)	1.7 ± 0.4	3.7± 0.2	6.0 ± 0.6	−5.7 ± 0.4
T_max_ (h)	2.5 ± 0.2	3.5 ± 0.3	3.7 ± 0.3	5.75 ± 0.4
% IOP reduction at T_max_	8.1 ± 1.7	20.1 ± 1.4	30.9 ± 2.5	27.6 ± 1.2
T_end_^f^ (h)	4.4 ± 0.6	24.0 ± 0.0	14.7 ± 1.3	>30
AUC ^g^ (%.h)	30.0 ± 7.1	196.7 ± 12.5	241.4 ± 14.6	645.2 ± 22.5

^a^ PRG: pregabalin. ^b^ EDF: enhanced delivery formulation. ^c^ Data are expressed as mean ± SEM; *n* = 6 rabbits, 12 eyes. ^d^ IOP: intraocular pressure. ^e^ T_max_: time to maximum response in hours. ^f^ T_end_: time to end of response in hours. ^g^ AUC (%.h): total area under % IOP reduction versus time curve.

**Table 10:** Statistical comparisons of PD parameters after application of a single dose of PRG ^a^-EDF ^b^, 0.6%, and marketed glaucoma medications to DB rabbits.

Pharmacodynamic Parameters	% IOP ^d^ Reduction at T_max_	T_end_^e^	AUC ^f^
Overall *p* value ^c^	<0.0001	<0.0001	<0.0001
Timolol maleate vs. latanoprost	0.0002 ^g^	<0.0001	0.0002
Timolol maleate vs. latanoprostene bunod	<0.0001	<0.0001	0.0001
Timolol maleate vs. PRG-EDF	<0.0001	<0.0001	<0.0001
Latanoprost vs. latanoprostene bunod	0.0041	<0.0001	0.6856
Latanoprost vs. PRG-EDF	0.0052	<0.0001	<0.0001
Latanoprostene bunod vs. PRG-EDF	0.5598	<0.0001	<0.0001

^a^ PRG: pregabalin. ^b^ EDF: enhanced delivery formulation. ^c^ Overall *p*-value represents the outcome of the one-way ANOVA analysis. ^d^ IOP: intraocular pressure. ^e^ T_end_: time to end of response in hours. ^f^ AUC (%.h): total area under % IOP reduction versus time curve. ^g^ Individual *p*-values represent the outcome of Tukey’s multiple comparisons test.

## Data Availability

The original contributions presented in this study are included in the article. Further inquiries can be directed to the corresponding author.

## Abbreviations

The following abbreviations are used in this manuscript:

PRG	Pregabalin
IOP	Intraocular pressure
EDF	Enhanced delivery formulation
Span 83	Sorbitan sesquioleate
SR PEG400	super refined polyethylene glycol 400
GCS	Glycol chitosan
DB rabbits	Dutch belted rabbits
PDI	Polydispersity index
BSS	Balanced salt solution
BCS	Biopharmaceutics classification system
WFI	Water for injection
EC_50_	PRG concentration corresponding to 50% of the highest response
T_max_	Time required to reach maximum decrease in IOP
T_end_	Time required for IOP to return to baseline value
AUC	Total area under the % IOP-vs-time curve
PD	Pharmacodynamics
PK	Pharmacokinetics
GRAS	Generally Recognized as Safe
AO	Acridine orange
LOQ	Limit of quantification
LOD	Limit of detection
